# NUPR1 acts as a pro-survival factor in human bone marrow-derived mesenchymal stem cells and is induced by the hypoxia mimetic reagent deferoxamine

**DOI:** 10.3164/jcbn.18-112

**Published:** 2019-03-23

**Authors:** Kazuhito Matsunaga, Koichi Fujisawa, Taro Takami, Guzel Burganova, Nanami Sasai, Toshihiko Matsumoto, Naoki Yamamoto, Isao Sakaida

**Affiliations:** 1 Department of Gastroenterology and Hepatology, Yamaguchi University School of Medicine, Minami Kogushi 1-1-1, Ube, Yamaguchi 755-8505, Japan; 2 Center for Regenerative Medicine, Yamaguchi University School of Medicine, Minami Kogushi 1-1-1, Ube, Yamaguchi 755-8505, Japan; 3 Institute of Fundamental Medicine and Biology, Kazan (Volga Region) Federal University, Kazan, Russia; 4 Department of Laboratory Science, Yamaguchi University School of Medicine, Minami Kogushi 1-1-1, Ube, Yamaguchi 755-8505, Japan; 5 Department of Oncology and Laboratory Medicine, Yamaguchi University School of Medicine, Minami Kogushi 1-1-1, Ube, Yamaguchi 755-8505, Japan

**Keywords:** mesenchymal stem cells, transcriptome, hypoxia, iron chelator, autophagy

## Abstract

Differences in the culturing conditions of mesenchymal stem cells used in regenerative medicine may affect their differentiation ability, genome instability, and therapeutic effects. In particular, bone marrow-derived mesenchymal stem cells cultured under hypoxia are known to proliferate while maintaining an undifferentiated state and the use of deferoxamine, a hypoxia mimetic reagent, has proven to be a suitable strategy to maintain the cells under hypoxic metabolic state. Here, the deferoxamine effects were investigated in mesenchymal stem cells to gain insights into the mechanisms regulating stem cell survival. A 12-h deferoxamine treatment reduced proliferation, oxygen consumption, mitochondrial activity, and ATP production. Microarray analysis revealed that deferoxamine enhanced the transcription of genes involved in glycolysis and the HIF1α pathway. Among the earliest changes, transcriptional variations were observed in HIF1α, NUPR1, and EGLN, in line with previous reports showing that short deferoxamine treatments induce substantial changes in mesenchymal stem cells glycolysis pathway. NUPR1, which is induced by stress and involved in autophagy-mediated survival, was upregulated by deferoxamine in a concentration-dependent manner. Consistently, NUPR1 knockdown was found to reduce cell proliferation and increase the proapoptotic effect of staurosporine, suggesting that deferoxamine-induced NUPR1 promotes mesenchymal stem cell survival and cytoprotective autophagy. Our findings may substantially contribute to improve the effectiveness of mesenchymal stem cell-based regenerative medicine.

## Introduction

Human bone marrow-derived mesenchymal stem cells (BMSCs) possess the ability to differentiate into adipocytes, chondrocytes, and osteocytes, and their application in regenerative medicine is highly anticipated. However, the culturing conditions may affect the quality of BMSCs and, thereby, their therapeutic performance. As the oxygen concentration in the bone marrow is about 1–7%, BMSCs cultured under 21% oxygen undergo stress, which may result in genetic instability. Repeated culturing may also result in genetic instability, cell aging, and decreased stemness maintenance. Interestingly, it has been reported that these problems can be overcome by hypoxic culturing.^(1–3)^ Furthermore, it is known that hypoxia preconditioning promotes mesenchymal cell proliferation and is utilized to increase MSC survival after transplantation.^(4,5)^ Although hypoxic culturing can be advantageous, it requires a specific equipment and cells may be temporarily exposed to non-hypoxia states during medium exchange. Thus, the possibility to employ hypoxia-mimetic reagents, such as deferoxamine (DFO), an iron chelator, has drawn much attention.^(6)^ Iron depletion by DFO mimics hypoxia by inhibiting iron-dependent prolyl-4 hydroxylase activity and, thereby, HIF1α hydroxylation and degradation. DFO preconditioning in Wharton’s jelly-derived neural-like cells has been shown to improve tolerance and therapeutic potential, and enhance the effectiveness of cell therapy.^(7)^ It has been reported that MSC preconditioning with DFO, before transplantation, increases the homing capacity of MSCs and endothelial progenitor cells^(8)^ and improves cell therapy.^(9)^ Moreover, hypoxic preconditioning potentiates the trophic and anti-apoptotic effects of MSCs on hepatocytes^(10)^ or brain cells.^(11)^ On the other hand, additional investigation is necessary to clarify the molecular basis of DFO effects. We have compared the changes in MSC metabolism induced by DFO and hypoxia, and found that low concentrations of DFO may be appropriate for MSC preconditioning.^(12)^

Nuclear protein 1 (NUPR1) (also known as p8) is a molecule that has recently caught much attention owing to its association with chemotherapy resistance in a wide range of cancer types. Its suppression of cell cycle via p53 and cytoprotective autophagy induction have been previously reported.^(13)^ To date, NUPR1 has mainly been studied in cancer cells, where it supports drug resistance. However, the underlying mechanisms have not yet been clarified in detail. Moreover, although an involvement of NUPR1 in the survival of mesenchymal stem cells has been speculated, no clues are available, to date, regarding its function in MSCs.

This study demonstrated the survival-promoting effect of treatments with low DFO concentrations for relatively short times (as low as 12 h) and investigated DFO-induced gene expression changes in MSCs. In addition, DFO-induced stress resistance mechanisms mediated by NUPR1 were explored. We expect that our findings will substantially contribute to the improvement of MSC survival and the advancement of MSC-based regenerative medicine.

## Materials and Methods

### Cell and cell culture

Bone marrow cells were purchased from (Lonza, Basel, Switzerland) and MSCs were cultured in DMEM medium, and cells at passages 3–8 were used. The MSC phenotype, CD11b(−), CD90(+), CD73(+), and CD105(+), was confirmed by flow cytometry.

### Proliferation assay

To measure proliferation, 1,000 MSCs were seeded in each well of a 96-well plate, DFO was then added at different concentrations, and the cellular area was evaluated with an IncuCyte HD imaging system (Essen BioScience, London, UK). DFO was purchased from Novartis Pharma K.K. (Tokyo, Japan).

### Apoptosis assay

After siRNA treatment, 3,000 cells were seeded in each well of a 96-well plate and cultured for 3 days. Next, cells were treated with staurosporine (0.3 µM) and 6 h later the cell number was measured with a CyQUANT^®^ cell proliferation assay (Thermo Fisher Scientific, Waltham, MA). Cells were then evaluated with a Caspase-3 Assay Kit (Promega, Tokyo, Japan) and caspase-3 activity per cell was measured. The results were obtained from three independent experiments with 6 replicates each.

### Western blot analysis

Western blotting was performed according to a standard method.^(14)^ In brief, cell lysis buffer contained 62.5 mM Tris-HCl (pH 6.8), 4% SDS, and 200 mM dithiothreitol. Cell lysates were electrophoresed on 12% acrylamide gels. Anti β-actin antibody (Sigma, Darmstadt, Germany), anti-p62 antibody (Abcam, Tokyo, Japan), anti-LC3 antibody (Life Technologies, Waltham, MA), and anti-NUPR1 antibodies (ProteinT, Chicago, IL) were used.

### Total RNA isolation

Total RNA was isolated from cells using TRIzol Reagent (Life Technologies) and purified using SV Total RNA Isolation System (Promega) according to the manufacturer’s instructions. RNA samples were quantified by an ND-1000 spectrophotometer (NanoDrop Technologies, Wilmington, DE) and RNA quality was confirmed with an Experion System (Bio-Rad Laboratories, Hercules, CA).

### SAGE

Ion Ampliseq Transcriptome Human Gene Expression Kit (Life Technologies) was used to create an Ion Proton next-generation sequencer library of analysis beads. An Ion PI IC 200 Kit (Life Technologies) and an Ion PI Chip Kit v2 BC were used for sequencing, using an Ion Proton next-generation sequencer. SAGE results were analyzed by Ingenuity Pathways Analysis (IPA).

### Oxygen consumption rate (OCR) measurements

 OCR measurements were performed using a Seahorse Biosciences XF96 Extracellular Flux Analyzer. Cells were seeded at 6,000/well in XF96 microplates (Seahorse Biosciences, Santa Clara, CA). After a 24-h incubation, the growth medium was replaced with XF Assay Medium (Seahorse Biosciences) supplemented with 25 mM glucose (Sigma-Aldrich, Darmstadt, Germany). OCR measurements were made over 5-min periods following a 3-min mix period. The following components were sequentially added to the cells: 1 µg/mL oligomycin (Sigma-Aldrich), 300 nM carbonylcyanide-*p*-trifluoromethoxyphenylhydrazone (FCCP; Sigma-Aldrich), and 2 µM rotenone (MP Biomedicals, Tokyo, Japan). The spare respiratory capacity and coupling efficiency were calculated according to the Seahorse Bioscience instructions and the basal OCR was normalized to the cell number.

### Immunofluorescence Analysis

MSCs were cultured in chamber slides, and then fixed in 4% paraformaldehyde. Immunofluorescence analysis was performed by using the Opal Multiplex Immunohistochemistry Kit (Perkin Elmer, Norwalk, CT) as per manufacturer’s instructions.

### Statistical analysis

The homogeneity of variance was assessed by the F-test. The results were analyzed by either the Student’s *t* test or Welsh’s two-factor *t* tests, and the data are presented as the mean ± SD, with the significance threshold established at *p*<0.05.

## Results

### DFO suppresses mitochondrial oxygen consumption and lowers the level of intracellular ATP

**Fig. 1:**
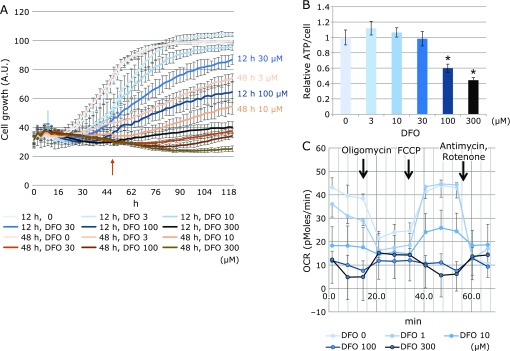
Evaluation of DFO effects on proliferation, ATP, and oxygen consumption of BMSCs. (A) A comparison of the effects of different DFO concentrations and treatment times on cell proliferation. In each well one thousand cells were seeded, DFO was added at different concentrations on the next day, and proliferation was evaluated every two hours with IncuCyte. DFO was administered for 12 h (brown profile) or 48 h (blue profile), and then the medium was replaced with DFO-free medium. The arrows indicate the termination time of DFO treatments (brown arrow, 12 h; blue arrow, 48 h). (B) The effects of DFO on ATP production. Three thousand cells were seeded in 96-well plates and, on the next day, ATP production was measured 12 h after DFO treatment. The ATP level was normalized based on the cell number deduced from CyQUANT^®^ cell proliferation assay (*p<0.05). (C) An evaluation of the OCR of cells after a 12-h DFO treatment using the Flux Analyzer. Six thousand cells were seeded in 96-well plates and OCR was measured on the next day, 12 h after DFO treatment. OCR was measured using the Flux Analyzer before and after the administration of oligomycin, FCCP, antimycin, and rotenone, as indicated.

In order to investigate the effects of DFO on cell proliferation, BMSCs were treated with different drug concentrations, for 12 and 48 h, followed by cell culturing in DFO-free medium. Proliferation was then evaluated by IncuCyte HD imaging system. We found that treatment for 48 h with 3 µM DFO, a concentration that is effective for medium preconditioning, and for 12 h with 50 µM DFO, resulted in comparable effects on cell proliferation (Fig. 1A). Next, the amount of ATP per cell was measured and found to be substantially reduced at DFO concentrations above 100 µM (Fig. 1B). To investigate the reason behind the ATP depletion, the OCR (oxygen consumption rate) was measured with a flux analyzer, revealing a detectable decrease of both basal and maximal respiration with 10 µM DFO, and a drastic reduction with 100 µM and 300 µM (Fig. 1C).

### DFO treatment alters the expression of genes for energy metabolism

**Fig. 2:**
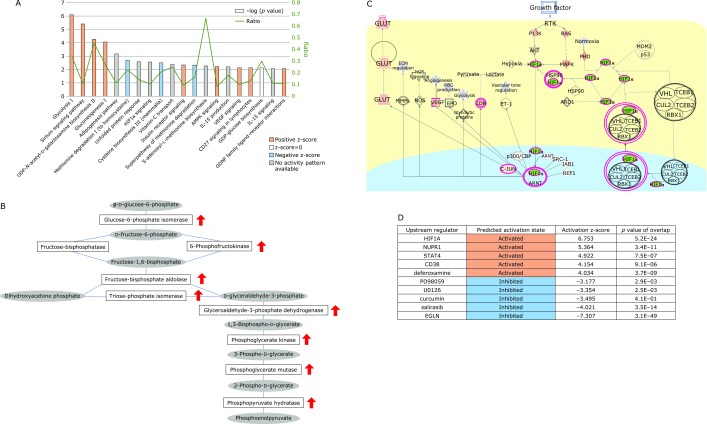
Analysis of DFO-induced gene expression changes. (A) Known pathway ranking through IPA. The bar graph shows the reciprocal display of *p* value calculated with the IPA software, while the green line indicates the proportion of genes included in each pathway. (B) Functional classification of gene clusters with altered expression (glycolysis). Red arrows indicate genes with a statistically significant upregulation in known pathway analysis (no downregulated genes were present in this data set). Rectangles indicate enzyme-encoding genes, while ovals indicate metabolites. (C) HIF1 signaling pathway analysis using IPA. The upstream and downstream effects of the mapped genes on HIF1 signaling pathway are indicated. Red and green symbols indicate genes up- and down-regulated in DFO treated MSC, respectively. The color intensity is proportional to the fold change. (D) Top 5 and bottom 5 upstream regulators. Brown indicates upstream regulators predicted to be activated, while blue indicates upstream regulators predicted to be inhibited.

After treatment of MSCs with 50 µM DFO for 12 h, RNA was extracted for microarray analysis. Functional classification of genes was performed using the IPA software, and pathways involved in sugar metabolism, such as glycolysis and gluconeogenesis, HIF1α-mediated and VEGF-mediated signaling, were classified as Canonical pathways (Fig. 2A). Examination of the glycolysis pathway revealed an upregulation of the enzymes glucose 6 phosphate isomerase, 6-phosphofructokinase, fructose-bisphosphate aldolase, triose-phosphate isomerase, glyceraldehyde-3-phosphate dehydrogenase, phosphoglycerate kinase, phosphoglycerate mutase, and phosphopyruvate hydratase (Fig. 2B). In the HIF1α pathway, due to HIF1α degradation inhibition by DFO, HIF1α was downregulated, and HIF1α target genes, namely GLUT, LDH and VEGF, were upregulated (Fig. 2C).

Among the upstream DFO-induced transcriptional changes, HIF1α was mostly activated, and hypoxia-induced EPAS1 and PDGF were also upregulated. Notably, NUPR1, which is known to be involved in mitochondrial stress, was activated. On the other hand, EGLN (hypoxia-inducible factor prolyl hydroxylase) and curcumin were among the suppressed upstream factors (Fig. 2D).

### DFO treatment enhances NUPR1 expression and autophagy

**Fig. 3:**
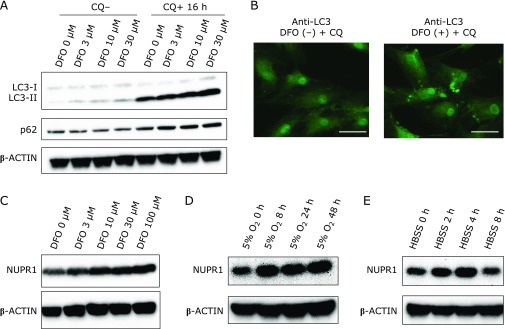
DFO-induced changes in NUPR1 expression and autophagy. (A) DFO-induced expression of autophagy-related proteins. Proteins were collected 16 h after the addition of chloroquine (CQ), and evaluated by western blotting. (B) DFO-induced autophagy visualized by immunohistochemical staining. The white bar indicates 50 µm. (C) DFO-induced changes in NUPR1 expression. (D) Hypoxia-induced changes in NUPR1 expression. (E) NUPR1 expression changes induced by cell culturing in amino acid-deficient medium (HBSS).

In a previously reported microarray analysis, we found that NUPR1 expression was increased in MSCs treated with 3 µM DFO for 48 h.^(12)^ Moreover, NUPR1 was found to be involved in cytoprotective autophagy. Thus, we examined the impact of DFO on autophagy. Interestingly, LC3-II was upregulated and p62 was slightly downregulated, and the amplitude of these effects was dependent on DFO concentration. Furthermore, both LC3-II and p62 were upregulated by DFO, in a concentration-dependent manner, after a 16-h treatment with chloroquine (CQ), an autophagy inhibitor, indicating the activation of autophagy (Fig. 3A). Furthermore, fluorescent immunohistochemical staining of cells treated with 10 µM DFO and CQ showed an increase in the LC3-positive puncta, suggesting DFO-induced autophagy enhancement (Fig. 3B). An analysis of NUPR1 expression revealed a DFO concentration-dependent increase 48 h after DFO treatment (Fig. 3C). In light of the observed DFO-induced NUPR1 upregulation, we next verified whether similar expression changes could be induced by hypoxia and found that these conditions also increased NUPR1 expression (Fig. 3D). In addition, enhanced NUPR1 expression was detected after a 2-h cell incubation with HBSS, an amino acid starvation medium known to enhance autophagy (Fig. 3E).

### NUPR1 knockdown reduces MSC proliferation and decreases drug resistance

**Fig. 4:**
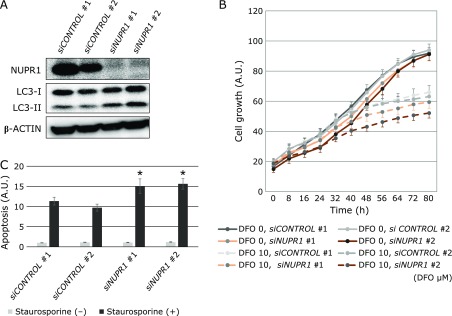
Changes induced by NUPR1 knockdown. (A) Western blot analysis in MSCs after siRNA-induced NUPR1 knockdown. (B) Effects of NUPR1 knockdown on MSC proliferation. Proliferation was measured every 2 h with IncuCyte (*n* = 6). (C) Effects of NUPR1 knockdown on staurosporine-induced apoptosis. Caspase-3 activity is shown 6 h after treatment with staurosporine was shown. Three thousand cells were seeded in 96-well plates and Caspase-3 activity was measured 6 h after staurosporine treatment. Caspase activity was normalized based on the cell number deduced from analysis with CyQUANT^®^ cell proliferation assay. * indicates statistically significant difference compared with *siCONTROL *#1, *p*<0.01.

Next, to directly explore the role of NUPR1 in MSC proliferation, its expression was suppressed by siRNA knockdown. The knockdown was efficient and resulted in clear LC3-II upregulation (Fig. 4A). Cell proliferation was slightly reduced in NUPR1-knocked down cells, but in the presence of DFO the decline became greater (Fig. 4B). Next, as NUPR1 has been reported to be associated with drug resistance in cancer cells, staurosporine-induced apoptosis was evaluated. NUPR1 knockdown increased caspase-3 activity in MSCs treated with staurosporine (Fig. 4C).

## Discussion

Previous reports have demonstrated that hypoxia preconditioning enhances MSC retention during intramuscular injection.^(7,9)^ We have also shown that DFO, a highly safe drug that has long been used to treat iron overload, can be an alternative to oxygen depletion as it mimics hypoxia.^(12)^ We found that relatively short incubations (12 h) with 50 µM DFO resulted in cell proliferation rates comparable to those observed after 48-h treatments with 3 µM DFO, i.e., the concentration normally used for preconditioning. Furthermore, ATP depletion, OCR decrease, inhibition of mitochondrial oxidative phosphorylation, as well as changes in glycolytic metabolism, were also observed after 12-h treatments. Therefore, we concluded that shorter preconditioning could be as effective as the conventional 48-h treatments.

It is thought that DFO suppression of cell proliferation occurs because HIF1α accumulates as a result of reduced PDH activity, required for HIF1α degradation. In the pathway analysis of gene expression, glycolysis was ranked first, because of the overexpression of 5 of the 6 enzyme-encoding genes involved in glycolysis. Moreover, sirtuin, HIF1α, and VEGF signaling pathways, involved in glycolytic and mitochondrial metabolism, were also ranked, thus confirming the presence of DFO-induced changes even after a 12-h treatment. DFO-induced variations occurred in a few upstream regulators. HIF1α and NUPR1 were upregulated, while EGLN and curcumin related genes were downregulated. As EGLN requires iron for enzyme activities, its inhibition upon DFO-induced iron deprivation was expected. It has been reported that curcumin possesses iron chelation ability^(15)^ and reduces iron in mice and rats.^(16)^ Moreover, its application as a suppressor of oxidative stress has been proposed.^(15)^ Although NUPR1 upregulation was an upstream effect of the DFO treatment, it was maintained after cell treatment with 10 µM DFO for 48 h.^(12)^ NUPR1 is thought to be involved in chemotherapy resistance in cancer cells and is induced by environmental stresses such as nutrient starvation and hypoxia. Moreover, NUPR1 is known to be involved in cytoprotective autophagy^(13)^ and possesses cell cycle suppression ability via p53. Therefore, the effects of DFO on autophagy were explored in MSCs. In fact, it was found that combined cell treatments with DFO and CQ increased the intracellular levels of LC3-II and p62, indicating autophagy enhancement. Moreover, NUPR1 was upregulated by DFO treatment, hypoxia, and amino acid starvation. By regenerating metabolic precursors and eliminating unwanted substances in the cell, autophagy functions as a survival mechanism that maintains cell functions under stress, and prevents cell death through organelle turnover.^(17)^ Although it has been reported that DFO treatment induces protein aggregation and aggresome in NIH3T3 fibroblasts, as well as the autophagic gene p62/SQSTM1,^(18)^ a report has indicated that DFO may suppress autophagy caused by iron overload.^(19)^ Thus, autophagy may result in different outcomes depending on environmental and/or physiopathological cell conditions. The possible involvement of NUPR1 in cytoprotective autophagy has drawn much attention.^(13)^ Here, its function was investigated by gene knockdown, resulting in inhibition of cell proliferation. This finding was consistent with the function of NUPR1 in MSCs as a pro-survival factor. However, the degree of this proliferation block was lower than the reported effects in cancer cells.^(20)^ The increase in LC3-II upon NUPR1 knockdown was consistent with a previous study reporting that NUPR1 is involved in the maintenance of the autolysosomal efflux in A549 cells and that NUPR1 depletion deregulates autophagic flux and impairs autolysosomal clearance.^(21)^ In addition, it has been reported that NUPR1 depletion leads to massive cytoplasmic vacuolization and premature senescence. However, the latter events were not observed in MSCs in this study (data not shown). As in these cells the block of proliferation induced by NUPR1 knockdown is less pronounced compared to cancer cells, MSCs might have a lower dependency on NUPR1 and phenotypes such as premature senescence might be less likely to occur. Further work will be necessary to solve these issues.

In this study, NUPR1 depletion enhanced staurosporine-induced apoptosis, indicating the importance of NUPR1 in drug resistance, in line with previously reported results.^(22)^ However, its role in drug resistance is yet to be clarified in detail. Pancreatic cancer cells have been reported to be resistant to starvation and gemcitabine treatment owing to the NUPR1/RELB/IER3 survival pathway, and CDKN1A/p21 phosphorylation and relocalization from the nucleus to the cytoplasm are important for breast cancer cell resistance to chemotherapeutic agents such as doxorubicin.^(23)^ In this study, the phosphorylation and localization of p21 were also evaluated but no clear changes were observed (data not shown). Recently, the usefulness of NUPR1 in cells other than cancer cells has also been studied, and it has been reported that, in keratinocytes, NUPR1 is involved in UV-induced stress response.^(24)^

Our findings, including the observation of glycolysis-dominant metabolic changes in MSCs treated with DFO for 12 h, suggested that it is possible to apply shorter times of hypoxic preconditioning, compared to traditional protocols. Furthermore, as DFO treatment, similarly to hypoxia and starvation, upregulates NUPR1, and this may lead to improved cell survival, our results may contribute to the design of optimized methods of MSC preparation for regenerative medicine.

## Author Contributions

Conceived and designed the experiments: KM, KF, TT and IS. Performed the experiments: KM, KF, TT, GB and NS. Analyzed the data: KM, KF and TT. Contributed reagents/materials/analysis tools: TM and NY. Wrote the paper: KM, KF and TT.
